# A new chapter in STI management: gepotidacin’s role in gonorrhea treatment

**DOI:** 10.1097/MS9.0000000000004841

**Published:** 2026-03-30

**Authors:** Zahabia Adnan, Hajra Rauf, Ahmed Asad Raza, Abedin Samadi

**Affiliations:** a Department of Medicine, Jinnah Sindh Medical University, Karachi, Pakistan; b Department of Medicine, Kabul University of Medical Sciences, Abu Ali Sina, Kabul, Afghanistan

**Keywords:** antimicrobial resistance, DNA gyrase inhibitors, gepotidacin, gonorrhea, sexually transmitted infections

## Abstract

The emergence of antimicrobial-resistant *Neisseria gonorrhoeae* has become a critical global health challenge, compromising the effectiveness of existing treatment regimens. Gepotidacin (GSK2140944), a first-in-class, orally administered triazaacenaphthylene antibiotic, offers a promising therapeutic alternative due to its novel dual-target inhibition of DNA gyrase and topoisomerase IV. This review summarizes current evidence on gepotidacin’s role in the management of uncomplicated urogenital gonorrhea. Findings from Phase II and Phase III clinical trials demonstrate microbiological cure rates exceeding 90%, comparable to the current ceftriaxone-based standard of care, with a favorable safety profile. Gepotidacin’s oral route, effectiveness against resistant strains, and utility for patients unable to receive cephalosporins or fluoroquinolones position it as a significant advancement in sexually transmitted infection management. Its introduction may strengthen antimicrobial stewardship, reduce complications, and enhance accessibility, particularly in low- and middle-income countries. Collectively, gepotidacin represents a major step forward in combating resistant gonorrhea and shaping future antibiotic development.

In a major advancement of infectious disease control, the US Food and Drug Administration (FDA) has approved gepotidacin (Blujepa), the first-in-class oral antibiotic which has emerged as a promising treatment for uncomplicated urogenital gonorrhea^[^1^]^. The global annual incidence of gonorrhea, affecting approximately 87 million adults, is recognized by the U.S Centers for Disease Control and Prevention (CDC) as a major public health threat^[^1^]^. It is a sexually transmitted disease caused by *Neisseria gonorrhoeae*, which has been classified by the World Health Organization as the global priority pathogen, due to its escalating drug resistance to nearly all classes of antibiotics^[^1^]^. The growing prevalence to both ceftriaxone and azithromycin resistance has weakened the effectiveness of standard therapeutic gonorrhea regimens. This concern of antimicrobial stewardship led the CDC to revise its 2020 guidelines, which recommends the discontinuation of azithromycin in combination therapy for the treatment of urogenital gonorrhea^[^2^]^. However, documented treatment failures of ceftriaxone have also emerged, which is currently the last reliable first-line option of treatment after 2020 CDC guidelines. This shows that persistence of multidrug-resistant and extensively drug-resistant *N. gonorrhoeae* strains represents a serious global health concern.

The global burden of reproductive and infectious diseases significantly increased due to inadequate management of gonorrhea, which leads to a wide range of complications. While the urogenital tract is a primary site of infection, it can also infect extragenital sites asymptomatically, such as the rectum, spinal cord, pharynx, and conjunctiva. The vulnerability to *N. gonorrhoeae* is most pronounced among sexually active adolescents, individuals with multiple partners, and people having limited access to preventive and sexual health services. One of the poignant examples highlights the spread of infection beyond the urogenital tract with the manifestations of arthritis, tenosynovitis, and skin lesions. Romiopoulos *et al* illustrated the devastating impact of *N. gonorrhoeae* in their report, which focused on a 41-year-old immunocompetent woman whose condition deteriorated as a result of the development of spinal abscess and spondylodiscitis from disseminated gonococcal infection^[^3^]^. Moreover, Swasdio *et al* elaborated a multicenter case control study on bilateral tubal occlusion in women with serological evidence of past gonococcal infection, which leads to ectopic pregnancy^[^4^]^.

Unlike most antibiotics that attack one point of bacterial invasion, gepotidacin is a first-in-class triazaacenaphthylene antibiotic that attacks two key enzymes, DNA gyrase and topoisomerase IV, required for bacterial DNA replication. The concurrent attack on two perpetrators is a first in this type of therapy. This dual target strategy is novel among known therapeutic agents. The FDA approval of gepotidacin is supported by data from both Phase II and Phase III clinical trials evaluating the efficacy against *N. gonorrhoeae*. The EAGLE-1 Phase III randomized, controlled, open-label trial compared oral gepotidacin of 3000 mg dosage, with the current standard regimen of ceftriaxone plus azithromycin, assessing approximately 400 patients with uncomplicated urogenital gonorrhea^[^5^]^. Gepotidacin showed 92.6% (187 out of 202) of microbiological success rates comparable to 91.2% (186 out of 204) rate in standard therapy. This shows the non-inferiority of the drug to ceftriaxone plus azithromycin with a well-tolerated safety profile. Earlier, Phase II, Randomized, Dose-Ranging study also showed similar promising results, with approximately 96% of microbiological cures in urogenital infections^[^6^]^. These findings laid the foundation for the subsequent Phase III evaluation.

Although gepotidacin’s dual inhibition of DNA gyrase and topoisomerase IV may increase the genetic barrier to resistance, selective pressure from widespread or empiric use could still promote reduced susceptibility. Experimental data suggest that clinically significant resistance may require concurrent target-site mutations; however, adaptive pathways can emerge under antimicrobial exposure, necessitating ongoing surveillance. Furthermore, existing clinical evidence is largely limited to uncomplicated urogenital infection, restricting extrapolation to extragenital reservoirs. Careful integration into antimicrobial stewardship programs will therefore be essential to preserve long-term effectiveness^[^5,7^]^.

Evidence for gepotidacin in special populations and extragenital infection remains limited. Pregnant individuals were excluded from pivotal clinical trials, and therefore its safety and efficacy in pregnancy have not yet been established. In a Phase II study, gepotidacin demonstrated high microbiological cure rates for uncomplicated urogenital gonorrhea (96%); however, evaluation of extragenital sites was limited, with cure observed in only one of two pharyngeal infections and three of three rectal infections, reflecting very small sample sizes^[^6^]^. This limitation is clinically relevant, as pharyngeal gonorrhea has historically been associated with lower cure rates and represents an important reservoir for ongoing transmission and antimicrobial resistance^[^8^]^. Further studies assessing gepotidacin in pregnancy and extragenital infections are therefore warranted before broader clinical application can be recommended.

Introduction of gepotidacin revolutionizes public health choices and prevents medical conditions other than urinary tract infections and gonorrhea, including fewer patients developing kidney infection, infertility, or spread of resistant organisms. Fewer complications translate into fewer admissions for hospital stay and less necessity for costly reserve drugs, which reduces overall expense. The drug enters surveillance networks from the outset, accompanied by the plans for stewardship strategies to ensure strict oversight and responsible use. Moreover, it is a drug that is effective in oral administration, which enhances patients’ adherence and broadens accessibility, especially in low- and middle-income countries (LMICs) where using injections may pose logistical challenges^[^9^]^. However, the practical impact of oral therapy in LMICs will also depend on affordability, procurement mechanisms, and the availability of diagnostic and antimicrobial resistance surveillance systems, to avoid inappropriate empiric use. In many resource-constrained settings, limited access to laboratory confirmation and test-of-cure practices may encourage syndromic management, increasing the risk of misuse of newly introduced oral agents. In the absence of robust surveillance and stewardship infrastructure, such patterns could undermine long-term effectiveness by accelerating the emergence and spread of resistance. Therefore, successful implementation will require alignment with existing public health programs, treatment guidelines, and surveillance networks to ensure responsible and sustainable use. Nevertheless, gepotidacin may provide a safer and effective alternative for patients with contraindications or allergies to cephalosporins or fluoroquinolones.

Collectively, gepotidacin not only represents a new era in sexually transmitted infection management but also provides a more effective and approachable model for future antibiotic innovation.

To ensure methodological transparency and ethical compliance in manuscript preparation, we adhered to the recently developed TITAN (Transparency in the Reporting of Artificial Intelligence) 2025 guidelines, which outline standards for responsible AI use in biomedical writing and publishing^[^10^]^.

## Data Availability

No datasets were generated or analyzed during the current study.
